# Characteristics of psychological time in patients with depression and potential intervention strategies

**DOI:** 10.3389/fpsyt.2023.1173535

**Published:** 2023-05-25

**Authors:** Hanlin Ren, Qing Zhang, Yanzhen Ren, Qiang Zhou, Yuan Fang, Liang Huang, Xiaobao Li

**Affiliations:** ^1^The Third People's Hospital of Zhongshan, Zhongshan, China; ^2^Fujian Key Laboratory of Applied Cognition and Personality, Minnan Normal University, Zhangzhou, China; ^3^School of Foreign Studies, Zhongshan Institute, University of Electronic Science and Technology of China, Zhongshan, China; ^4^Faculty of Education, Henan University, Kaifeng, China

**Keywords:** depression, psychological time, time perception, time perspective, circadian rhythms, passage of time

## Abstract

Psychological time reveals information about an individual’s psychological state and psychopathological traits and, thus, has become a new perspective through which the occurrence and development of depression can be examined. Psychological time includes time perception, time perspective, circadian rhythms, and passage of time. Patients with depression are characterized by inaccurate time interval estimation, habitual negative thoughts about the past and future, evening-type circadian rhythms, and slow passage of time. Habitual negative thoughts about the past and future and evening-type circadian rhythms influence the formation of depression, and poor time interval estimation and slow passage of time may result from depression. Further study is needed accurately exploring psychological time and influencing factors in patients with depression, and prospective cohort studies could further clarify this complex relationship. In addition, the study of psychological time has important implications for developing effective interventions to reduce depression.

## 1. Introduction

Major depression is a serious and common psychopathological disorder that has severe affective, cognitive, and behavioral consequences. According to the World Health Organization (1), more than 300 million individuals have depression worldwide; depression represents the fourth highest disease burden and is the leading cause of functional disability, affecting families and society. At present, the etiology of depression is not clear. The causes of depression have been explored from the perspectives of genetics, adverse events, and cognitive patterns. Among them, time is an important perspective of depression diagnosis and treatment (2, 3).

Since von Gebsattel, Minkowski and others (4–6), time has been a long-standing theme of phenomenological psychopathology and of phenomenological philosophy as time constitutes the bedrock of any experience. Drawing on philosophical concepts of Bergson, Husserl, and Heidegger, these authors have analyzed psychopathological deviations of time experience mainly from an individual point of view (7). In addition, researchers have used empirical qualitative studies, content analysis, and interviews to explore the abnormal time experiences of people with psychiatric disorders (e.g., schizophrenia, depression, mania, obsessive–compulsive disorder, etc.) (8, 9). It turns out that abnormalities in time experience are the basic dysfunction underlying the various symptoms in the different domains (10). In addition, phenomenological psychopathology, through theoretical and idiographic studies, conceptualizes depressive disorder as a disorder of time experience (8). In other words, disturbed of psychological time in patients with depression.

Psychological time is an individual’s subjective representation of clock time (11), and it mainly includes time perception, time perspective, circadian rhythms, and passage of time (12). First, time perception is the way in which the human brain understands the continuity and order of things or events (13). Second, time perspective is a personality trait that refers to an individual’s cognition, experience, and action (or tendency to act) in relation to time (14). Third, circadian typology refers to individual circadian rhythms from a biological perspective, and individuals can be categorized into three circadian rhythm types: evening, morning, and neither. Finally, passage of time is defined as an individual’s impression of time passing quickly or slowly (11).

Psychological time is significant in the diagnosis and treatment of various mental disorders, as time perception and individual time represent mental space–time organization; understanding these mechanisms is not only beneficial to the diagnosis and evaluation of patients with mental disorders but also to the improvement of mental disorder treatment and rehabilitation (2, 3, 15–18). Although researchers have begun to examine the internal relationship between psychological time and depression (19–22), no unified conclusion has been formed, and no systematic summary of this work has been conducted. Consequently, the current review summarizes the characteristics of psychological time in patients with depression, elaborating on time perception, time perspective, circadian rhythms, and passage of time, and exploring the internal mechanisms relating psychological time and depression. This study aimed to provide a theoretical basis for scientific research in depression-related fields and to highlight potential clinical interventions.

We searched for relevant studies in PubMed/Semantic Scholar/CNKI. The primary key words were “depression” and “timing” or “time” or “circadian rhythms.” Additional studies were identified by including the references listed in the studies found in PubMed/Semantic Scholar/CNKI, and by considering the studies that cited the resulting body of literature, again using in PubMed/Semantic Scholar/CNKI. The literature covers a period from 1928 to 2023. It focuses mainly on empirical research on psychological time in patients with depression (including four aspects), and includes several research reviews. And a small number of non-depressive studies closely related to psychological time.

## 2. Psychological time characteristics in patients with depression

### 2.1. Time perception

Time perception has a fundamental effect on personal function, and it can be characterized as the matching or mismatching between clock and experience of time. Time perception mainly includes succession (i.e., identification of time order) and duration (i.e., identification of event persistence) (11). Existing studies on time perception in patients with depression have mainly focused on judgment of time duration, as this is affected by emotion and attention (19). Indeed, studies have shown that, compared with healthy individuals, patients with depression may show inaccurate time interval estimation. For example, Mioni et al. (23) adopted the time production task, in which the experimenter specifies a time interval in temporal units and the participant reproduces this interval. Patients with depression tended to overestimate intervals of 500 ms and underestimate intervals of 1,000 and 1,500 ms. Liu et al. (24) used the temporal bisection task, in which participants judge the similarity of presented intervals, and found that patients with depression tended to overestimate intervals of 400–1,000 ms and underestimate those of 1,600 ms. Similarly, Tao et al. (25) found that both a group with clinical depression (meeting the diagnostic criteria for clinical depression) and a group with subthreshold depression (a depressive state with symptoms of depression that does not meet the diagnostic criteria for depression) tended to overestimate durations of 400 ms and underestimate durations of 1,600 ms.

In terms of the estimation of longer durations (35, 90, and 109 s), patients with depression tend to underestimate durations (21). Therefore, patients with depression may overestimate short durations (less than 1 s) and underestimate long durations (1 s and above) (2, 25). However, both Oberfeld et al. (26) and Hawkins et al. (27) did not find abnormal time perception in patients with depression. However, in the study by Oberfeld et al. (26), the sample size was small, and controls were not age- and sex-matched. The participants in a study by Hawkins et al. (27) were study were healthy individuals in whom depression was induced; therefore, the results may have differed from the true characteristics of time perception in patients with depression. However, the meta-analysis by Thones and Oberfeld et al. (28) showed no significant effects of depression in the four time perception tasks. Except in time production, there was a tendency towards overproduction of short and underproduction of long durations in depressive patients compared to healthy controls task. Therefore, we should continue to explore the characteristics and influencing factors of time perception in patients with depression and reach a consistent conclusion as soon as possible.

Studies have shown that an increase in dopamine levels can lead to the overestimation of duration, whereas a decrease in dopamine levels can lead to the underestimation of duration (28, 29). Dopamine levels are associated with arousal (30–32) and attention (33, 34). Due to disordered dopamine levels in patients with depression (28), at the beginning of time tasks, patients with depression may be in a higher state of attention and arousal, and dopamine levels may be higher, thus may resulting in the overestimation of short durations. In addition, electroencephalography has shown that amplitudes at LPCt electrodes were negatively correlated with duration judgment; specifically, smaller amplitudes were associated with judgments of a duration as long (35). Compared with healthy individuals, patients with depression have lower LPCt amplitudes when judging 400-ms intervals, thus resulting in an overestimation of short durations (25). Moreover, the basal ganglia and cerebellum play important roles in the evaluation of short durations; abnormal activation in patients with depression may lead to the overestimation of short durations.

Due to the attention deficits observed in patients with depression (36), their arousal and attention levels may decrease rapidly as the task progresses, which could lead to a decrease in dopamine levels and, thus, may underestimate long durations. In addition, cognitive factors, such as attention and memory are particularly important in judgments of duration of more than 1 s (28). Patients with depression have dysfunctions in the frontal network region responsible for attention control, which may contribute to their reduced sensitivity when attending to long durations (37). Moreover, working memory plays an important role in duration information processing at 1,500 ms, and the working memory of patients with depression is decreased compared to that of healthy controls (34). Furthermore, negative cognition is often activated in the working memory of patients with depression during time tasks, especially in the case of long durations, thus disrupting time processing; Indeed, this phenomenon may be an important reason for the underestimation of long durations (37). And, abnormal changes in the parasympathetic nerve (38) and abnormal gray matter volume (39) can also lead to inaccurate time estimation.

Overall, inaccurate duration estimation in patients with depression may be related to abnormal neural activation and deficits in attention and executive function, which are required for time processing (2, 28, 40). Due to the short times involved in duration estimation, these estimations are easily affected by the external environment, experimental method, and emotional state of the individual (2, 15, 16, 28). Therefore, future studies should identify confounding variables, further explore the processing of different time durations in patients with depression, examine the roles of dopamine and various brain regions, and clarify the influence of other cognitive factors in addition to attention and memory on time perception.

### 2.2. Time perspective

Time perspective (TP) is a personality trait referring to an individual’s cognition, time experience, and action (or tendency to act) in relation to past, present, and future time (14). Time perspective plays a role in past, present, and future time frames (12, 41, 42), meaning it can be divided into past time perspective, present time perspective, and future time perspective (14). At present, researchers mainly use the Zimbardo Time Perspective Inventory to measure the characteristics of individual time perspectives. Zimbardo and Boyd (41) divided time perspective into five dimensions: past positive (love of one’s past), past negative (negative evaluation of the past), present hedonistic (enjoyment of the present moment), present fatalistic (fate out of one’s control), and future (preparation for the future). Furthermore, Zimbardo and Boyd (41) have introduced the idea of Balanced Time Perspective (BTP), defining it as “the mental ability to switch effectively among TPs depending on task features, situational considerations, and personal resources.” Individuals displaying low scores in Past Negative and Present Fatalistic, moderate scores in Present Hedonistic and high scores in Past Positive and Future are considered to reflect the BTP profile (43). To measure the BTP profile one coefficient has been developed: the Deviation from the Balanced Time Perspective (DBTP) coefficient, and the farther a DBTP value is from zero, the more misbalanced an individual’s TP profile is considered (44). Therefore, researchers often use DBTP to measure whether an individual’s TP is balanced and use each TP to measure the characteristics of an individual’s time perspective (45–48). Numerous studies have shown that time perspective is related to an individual’s emotional state, academic performance, achievement motivation, subjective well-being, and career development (49, 50). It has special significance in the treatment of various mental disorders (3, 17).

Depression is one of the most common mental disorders, and patients with depression have unbalanced TP. TP is a relatively stable personality trait and takes a long time to form (14), and depression follows a process from initial development to diagnosis, making it difficult to determine causal relationships between these factors. Therefore, existing studies have focused on correlations between depression and TP or unbalanced TP in patients with depression. Several studies have reported that patients with depression have greater DBTP (51, 52), the greater DBTP was associated with somewhat higher symptoms of depression (53), lower DBTP predicted greater task engagement, lower worry, distress and depression (48). To sum up, the most consistent results are presented for links between greater DBTP and distress, as well as symptoms of mood problems (48).

In terms of the five dimensions of time TP, the most prominent feature of patients with depression in terms of TP is the past negative; They hold a serious pessimistic attitude to the past, focus on past negative experiences, and are unable to extricate themselves from the past (17, 54, 55). Empirical studies have shown that past negative is more correlated with depression than other time dimensions (16, 41). In comparison with the combined pattern scores of TP(e.g., deviation from a negative score or balanced TP scores), past negative is more strongly correlated with depression (18). Moreover, a previous study examining sandplay therapy in patients with depression found a significantly higher number of negative situations than positive ones; the negative situations where most commonly in the past, followed by those in the future (56). Future negative is the second most important feature of time perspective in patients with depression. In a previous study, participants were asked to rate the likelihood of certain events happening to them and how they would react to those events (such as learning well or a break-up). The group with depression overestimated the probability of negative events and predicted a lower intensity of happiness after positive events (57). Moreover, individuals with depression not only tend to predict negative events in the future but also believe that future events were likely to produce negative outcomes (58). In addition, present fatalistic is another characteristic of individuals with depression. Specifically, fatalism refers to an individual not believing in the possibility of change and tending to blame their problems on fate or external forces (18, 59). In terms of other time dimensions, patients with depression have often been observed to not be able enjoy their current life and are pessimistic about their current situation (20). In summary, patients with depression have the characteristics of past negative, present fatalistic, and future pessimistic, as shown in Table 1.

**Table 1:** Characteristics of time perspectives in patients with depression.

Dimension	Characteristics
Past negative	Focus on negative events in the past
Past positive	Thought of nothing to miss about the past
Future	Thought of the future being hopeless and negative
Present fatalistic	Thought of inability to change present situation
Present hedonistic	Inability to enjoy the moment and make oneself happy

Current research suggests that unbalanced TP may play an important role in the development of depression. For example, Micillo et al. (60) investigated the relationship between depression and TP during the first few weeks of the COVID-19 pandemic and found that unbalanced TP was the main predictor of increases in the degree of depression and an important predictor of depression levels. Additionally, Åström et al. (45) found that past and future negative were significant predictors of depression in older adults. Gu (61) found that negative TP played an important mediatory role in the relationship between family function and depression in adolescents. Although these studies all confirmed the important effects of TP on depression, most used cross-sectional designs and, thus, could not support a causal relationship between TP and depression. Indeed, depression is often assessed based on the symptoms experienced in the prior 2 weeks (62), and TP is a stable personality trait (14). Therefore, it is questionable as to whether patients’ depressive symptoms in the previous 2 weeks could lead to changes in their stable TP. In addition, most patients with depression have personality traits that make them vulnerable to depression (63, 64). The development of depression is a process; however, whether TP changes before the diagnosis of depression remains unknown. Overall, depression and unbalanced TP may have a bidirectional relationship. Negative perceptions of the past and future may contribute to depressive feelings, and depressive feelings may affect TP.

### 2.3. Circadian rhythms

Circadian rhythm is an endogenous life process with approximately 24-h cycle fluctuations (65). Circadian rhythms reflect individuals’ preferences for different time periods, and based on this, individuals can be divided into evening types, morning types, and neither type (66). Studies have shown that circadian rhythm disorders are significantly associated with depression. For example, workers alternating between day and night shifts have more depressive symptoms than those who work day shifts. Individuals who go to bed late and sleep for short periods are at higher risk of depression among day-shift workers (67). Moreover, late sleeping is an important predictor of the development and increase in depressive moods (68), and early rising can effectively relieve the symptoms of depression (69). Therefore, being an evening type is a risk factor for depression, whereas being a morning type is a protective factor (70).

Studies have shown that the increase in melatonin at night is key for individuals to maintain good sleep (71, 72). Melatonin follows a diurnal cycle, with low levels during the day, a rapid increase at night, a peak in the middle of the night, and then a gradual decline (72). However, night-time light exposure reduces melatonin secretion, causing difficulty falling asleep and decreased sleep quality, thus, weakening emotional regulation ability. This effect, in turn, can result in depression, anxiety, and other problems (73, 74). In contrast to the increase in melatonin in the evening, cortisol reaches its lowest level in the middle of the night in order for the body to recover (75).

Cortisol is closely related to the processing of stressful events. Under the stimulation of stressors, cortisol levels rise rapidly to allow the individual to adapt to certain environmental changes (76). However, sleeping late leads to a high level of cortisol at night (77). In the long term, this may lead to a decline in immunity, an imbalance of the stress regulation system, difficulty in emotional regulation, and, thus, emotional disorders (78, 79). Previous studies have confirmed that cortisol levels is an important indicator of the risk of depressive symptoms and are an important predictor of depression (80, 81). In addition, late sleepers show more amygdala activity at night and lower functional connectivity between the amygdala and the cingulate gyrus. This lack of regulatory inhibition on the amygdala may mean that evening types produce more intense emotional responses when faced with negative stimuli, thus affecting emotional regulation (82). In conclusion, the internal system disorder caused by late sleeping may be an important reason evening-type individuals are prone to depressive symptoms.

Although the above studies demonstrate the predictive role of circadian rhythm on depressive mood from a physiological perspective, the causal relationship is not clear (66). Factors associated with depression that occur concurrently with certain sleep rhythms, such as personality traits, lifestyle, and family relationships, add to the uncertainty regarding causality. In the future, more rigorous prospective cohort studies should be designed to further determine the relationship between depression and circadian rhythms (65).

### 2.4. Passage of time

Passage of time is defined as an individual’s impression of time passing quickly or slowly (12). An association between passage of time and time perception, both of which reflect individuals’ subjective feelings towards clock time. However, passage of time is a judgment about how fast time seems to pass in certain situation. It is generally measured with a question such as “How fast does time pass for you?” or “How did time pass relative to clock time?” (83). Time perception is a duration judgments, being assessed by verbal estimation and interval production measures (83). In addition, we previously studied other terms that are similar to the “individual’s impression of time passing quickly or slowly” (8, 84). However, the differences among these terms are beyond the scope of this article.

Due to the influence of many factors such as emotion and attention, individuals’ experience of time differs from physical time (24). Among the factors that influence passage of time, changes in mood may have the greatest impact (85). Depression is an emotional disorder that changes an individual’s experience of time (37). Indeed, it has been found that many patients with depression usually report that current time passes slowly (37, 56, 86). For example, patients with depression may report that “Time seems to have stopped” or “Time is dreadfully slow” (19). In addition to these oral reports, some empirical studies have been conducted to prove that individuals with depression experience slower passage of time. For example, Hofer and Osmond (87) asked patients with depression to judge their agreement with certain statements, such as “time seems to pass slowly, “and found that most patients with depression felt that time passes more slowly than healthy controls. Kitamura and Kumar (88) and Bschor et al. (89) used a self-rating questionnaire of passage of time and a visual analog scale, respectively, used to study the passage of time in patients with depression. These studies found that patients with depression felt the time passing slowly, and the perceived slowness was positively correlated with the severity of depression. Therefore, time retardation is the main feature of passage of time in patients with depression.

Studies have shown that the speed of passage of time is not only closely related to emotions but also to attention, time pressure, and vitality (90, 91). When individuals are in positive emotional states, such as joy and happiness, they feel that time passes quickly. However, when individuals are in negative emotional states, such as sadness and pain, they feel that time passes slowly (91). As patients with depression are often in a negative emotional state, they experience the feeling of time passing slowly. When individuals focus their attention on time information, such as by watching the clock repeatedly, they also feel that time is passes slowly (92). Indeed, patients with depression lack interest in daily life and pay too much attention to time changes; therefore, they are more likely to have the feeling of slow time passage (37). Moreover, with lower time pressures, individuals are more likely to perceive time passing slowly (93), and time pressure is related to the urgency of task completion (94).

Patients with depression show less motivation and confidence to complete tasks and low time pressure, which may produce feelings of low time passage slowly. In addition, time is perceived to pass slowly with lower individual vitality. For example, the sense of time passing in older adults is slower than in young individuals (90). The slow movements and low mental vitality of patients with depression may also be an important reason for their slow passage of time. In conclusion, depressive symptoms may be the main reason for the slow passage of time in patients with depression. Slow passage of time is a mark of subtypes of depression (9). However, existing studies have mainly focused on the description of phenomena or behavior analysis, and research on the neural mechanisms of passage of time is lacking. In the future, behavioral and neuroscience studies should be combined to further elucidate the factors affecting passage of time and the important roles played by each brain region in passage of time.

### 2.5. The relationship between the four aspects of psychological time

Research has shown that TP is closely related to circadian rhythms. For instance, Beracci et al. (95) found an association between present hedonism and evening-type and future and morning-type. More BTP individuals exhibited more pronounced morning preference (48). Moreover, these two aspects tend to be personal traits, which can affect time perception and passage of time (14, 96–100). Time perception and passage of time both reflect the subjective feeling of time. Time perception is the judgment of time interval, whereas passage of time is a judgment of how fast time seems to pass in certain situation (83). Both may be stated to be vulnerable to the influence of personality traits. For instance, Wittmann et al. (96). found that present hedonism is linked with a faster passage of the last week; the past negative perspective is related to time pressure; a future perspective is related to a general faster passage of time. And, individuals experience the passage of time differently at different time periods (97). Specifically, the internal clock ran relatively faster when the circadian oscillation of body temperature was on the rise and relatively slower on the declining portion of the temperature curve (98).

In addition, TP and circadian rhythm affect time perception. For example, Witowska (99) found that BTP was associated with a more accurate perception of time, and individuals with high DBTP were less accurate in the duration reproduction task. Kuriyama and Uchiyama (100) found that circadian rhythm affects the accuracy of individuals’ time production task through core body temperature and serum melatonin levels. These results suggest that human time perception may be more influenced by circadian rhythm than working memory load or psychophysiological status (100). In summary, we can infer that TP and circadian rhythm influence each other and both lead to changes in individual time perception and passage of time. However, the above studies are mainly related studies, and the causal relationship between psychological time still needs to be further verified and explored.

## 3. Model of the relationship between psychological time and depression

Based on this review of psychological time in patients with depression, it is clear that various factors influence psychological time and depression. With reference to the results of previous studies, a relationship model was developed between psychological time and depression in this study. First, biological, social, and psychological factors are not only important causes of depression (63, 64) but may also influence unbalanced TP and circadian rhythm (69, 101, 102). For example, individuals with high levels of childhood trauma tend to develop unbalanced TP (102). Additionally, chronic stress can lead to insomnia, dreaminess, circadian rhythm disturbance, and other problems (103). Secondly, psychological imbalances and physiological system disorders caused by unbalanced TP and circadian rhythm disorders are important factors in development of depression. For example, negative tendencies toward the past, present, and future affect the individual’s enthusiasm and hope for life, which may lead to depression (102). Furthermore, long-term late sleeping leads to issues with the secretion of melatonin and cortisol, affects the emotional control and regulation system, and makes individuals susceptible to depression and anxiety (22).

Finally, poor time estimation and slow passage of time may be due to depression. Both time perception and passage of time relate to an individual’s subjective perception of clock time. The former focuses on the perception of time intervals, whereas the latter relates to the overall perception of the speed of current time passage. Both time estimation and passage of time may have the same neural basis, with the involvement of areas, such as the insula, amygdala, and basal ganglia. Patients with depression have abnormal responses in these regions in relation to time estimation and experience (19, 21). In addition, for healthy individuals, inaccurate time interval estimation and a slowing of passage of time occur temporarily when they are affected by emotion, attention, or other factors (24, 90). Depression and attention disorders are among the main manifestations of depression. Therefore, abnormal neural mechanisms and depressive symptoms may be the main causes of abnormal time perception and passage of time in patients with depression.

The model may be bidirectional, as depression has important effects on TP and circadian rhythm. For example, bad experiences that individuals with depression cannot ignore may lead to fatalism in the present and a sense of hopelessness in the future (17). Moreover, problems, such as poor sleep quality and circadian inversion, in patients with depression may play a role in the disturbance of circadian rhythm (71). This model represents the attempt to clarify the complex relationship between psychological time and depression, revealing the internal mechanisms. This has theoretical and practical value for the prevention and treatment of depression. However, many current studies do not distinguish between the severity and age of patients with depression, which are important factors that affect individuals’ psychological time (59). In the future, it should be further verified whether the relationship between depression manifestations and psychological time of patients with different degrees of depression and different ages conforms to this model.

## 4. Intervention

Based on the discussion of the relationship model between psychological time and depression in the previous section, TP is found to be closely related to circadian rhythm, both being important factors leading to the development of depression. Depression aggravates the unbalance in patients’ TP, exacerbate the degree of circadian rhythm disturbance, and affect patients’ perception of time and passage of time. Therefore, depressive symptoms may possibly be improved by intervening with TP and circadian rhythm. Concurrently, both physical and medical treatments can be used to treat depression and improve patients’ inaccurate time estimation and slow passage of time. A more balanced TP leads to a better mental state of individuals and those of the morning type (48, 95). Therefore, time intervention is not only effective in the treatment of depression but also plays an important role in promoting the treatment of other mental diseases and maintaining mental health.

Time plays an important role not only in the treatment of depression but also in the psychotherapy of other mental illnesses. Some psychotherapy approaches focus on time to solve problems. Typical examples are mindfulness. Being in the present moment is a key element in most widespread definitions of modern mindfulness. A claim about temporality can thus be said to lie at the core of mindfulness (104). Using the example of mindfulness-based cognitive therapy (MBCT), designed to prevent relapse in recurring depression, mindfulness emerges as an ‘instantly effective’ intervention; as a discipline to be practiced for one’s future health; and as a perennial property of human attention, to be experienced in any present moment (105). Time perspective is a time-based therapy that is used to help the patient establish a balance among the past, present, and future to form a balanced TP (106). In addition, In addition, narrative therapy helps individuals establish a connection between the past, present, and future by dealing with the problems encountered from different angles and forming stories (107).

### 4.1. Inaccurate time estimation intervention

Due to the important role of the frontal cortex in individual time perception (108), some researchers have used repeated transcranial magnetic stimulation (rTMS) on the left and right dorsolateral prefrontal lobes to improve abnormal time perception in patients with depression. TMS is a non-invasive technique that uses pulsed magnetic fields to affect the activity of brain regions *via* the induction of a current. Jin et al. (109) compared the difference between medication and medication combined with left dorsolateral prefrontal rTMS therapy. After 4 weeks of treatment, depression levels decreased significantly in both groups, but only those patients with combined medication and rTMS showed significant improvements in time perception accuracy. Moreover, rTMS of the right dorsolateral prefrontal lobe can reduce patients’ errors in time perception (110). However, few of such studies exist, and the sample sizes were small. In the future, the sample sizes should be increased to further explore the effectiveness of this technique in improving abnormal time perception in patients with depression.

### 4.2. Unbalanced time perspectives intervention

Time perspective therapy is a therapeutic method based on time perspective (106). The concept of this therapeutic method is that time perspective affects a person’s emotions, thoughts, and behavior. Helping patients identify good things that have happened, take note of present good experiences, and actively plan for the future can re-establish a balanced perspective of time, which is compassionate to the past, more satisfied with the present, and more positive about the future (111). Empirical studies have shown that time perspective therapy can not only effectively promote patients’ recovery to health and reduce depression and anxiety (112) but can also play an important role in reconstructing time perspectives, reducing depressive symptoms, and enhancing life meaning in patients with comorbid cancer and depression (113). However, the popularity of time perspective therapy is not high, and there are few empirical studies have focused on this method. In the future, the characteristics of individuals across various countries and backgrounds should be considered to optimize and explore more suitable methods of time perspective therapy for different ethnic groups.

### 4.3. Circadian dysregulation intervention

At present, the main methods to ameliorate disorders of the circadian rhythm are exogenous melatonin therapy and light therapy. Exogenous melatonin therapy is effective quickly but has significant side effects, whereas light therapy is an effective and safe treatment that has slow onset but few side effects (114). In principle, light therapy affects the suprachiasmatic nucleus, which influences circadian rhythms *via* the hypothalamic tract, controlling the timing of the biological clock and melatonin secretion. Specifically, the suprachiasmatic nucleus controls awakening or sleep *via* circadian regulation of melatonin secretion (115). In a meta-analysis of phototherapy for sleep problems involving 53 studies with a total of 1,154 participants, light therapy was found to be highly effective for sleep dysrhythmia and insomnia (116). However, light therapy courses take a long time and require a certain amount of time and energy from the individual. In addition, light therapy is commonly investigated in research, but its practical application is not common. In the future, the research and exploration of this therapy should be strengthened.

### 4.4. Intervention for the slow passage of time

Although the characteristic of slow time passage in patients with depression has long been known by researchers (19), no targeted intervention method is currently available. The reason could be due to the influence of emotions on slow time passage. Indeed, for healthy individuals, passage of time changes with emotional fluctuations (56). Due to the close relationship between slow time passage and depressive mood (37), we infer that drugs that improve depressive mood may ameliorate the feature of slow time passage. In the future, more effective drugs should be developed to treat depression, which will not only improve depression but also solve the problem of slow time passage.

## 5. Summary and future prospects

### 5.1. Prospective cohort study

Based on this review of psychological time in patients with depression, the relationship between psychological time and depression is not clear. Currently, cross-sectional studies limit the inference of causality, and this is further affected by differences in patient characteristics, experimental paradigms, and technical means. Future prospective cohort studies should be designed to identify confounding variables that may have important effects on both depression and psychological time to better understand the relationship between both factors. Concurrently, the processing mechanisms underpinning the characteristics of psychological time in patients with depression should be investigated by combining disciplines, such as sociology, psychology, genetics, zoology, psychiatry, and psychosomatic medicine, etc. This information would help to clarify the relationship between depression and psychological time and provide more effective objective indicators for the identification, screening, and treatment of patients with depression.

### 5.2. Exploring the characteristics of psychological time across different subtypes of depressions

Based on severity, depression can be divided into mild, moderate, and severe depression. Furthermore, depending on the presence of other mental disorders, depression can be divided into simple depression and depression comorbid with other mental disorders, such as anxiety. Depression can also be classified into first-episode depression and recurrent depression (117, 118). Different types of patients with depression have different clinical manifestations, and their psychological time may also have different characteristics. However, a comparative study is lacking. Therefore, further distinguishing and exploring the psychological characteristics of different subtypes of depression would be of great significance for the formulation of targeted treatment programs.

### 5.3. Early detection and intervention based on abnormal psychological time

As psychological time is closely related to depression, increasing the understanding of psychological time in patients with depression, their families, educational staff, and healthcare personnel can help achieve early detection and intervention. Currently, medication-assisted psychotherapy is often used in the treatment of depression. In long-term treatment, patients can easily become dependent on medication. Working on their abnormal psychological time could effectively improve the mental state of patients with depression (112). However, few interventional studies have been conducted on psychological time in patients with depression. Therefore, more research in this area is particularly important for future treatment of patients. Concurrently, cognitive behavioral therapy, psychoanalysis, mindfulness therapy, VR technology, and drug therapy can be comprehensively used to improve patients’ depressive symptoms and abnormal psychological time.

### 5.4. Cross-domain comparison and specification of terminology

Since the research content of time psychology is relatively complex and interdisciplinary, challenges, such as the mixed use of terms, prevail. Therefore, this study only explored the psychological time characteristics of patients with depression but did not compare different research fields or distinguish mixed terms in detail. In the future, interdisciplinary comparisons between different fields of time psychology and standardized terminology should be developed.

## Author contributions

HR and QZhang designed the research and wrote the manuscript. YR, QZhou, and YF collected the data. XL and LH critically reviewed the manuscript and approved the final manuscript. All authors contributed to the article and approved the submitted version.

## Funding

This study was supported by grants from the Zhongshan Social Welfare and Basic Research Project (2022B1062).

## Conflict of interest

The authors declare that the research was conducted in the absence of any commercial or financial relationships that could be construed as a potential conflict of interest.

## Publisher’s note

All claims expressed in this article are solely those of the authors and do not necessarily represent those of their affiliated organizations, or those of the publisher, the editors and the reviewers. Any product that may be evaluated in this article, or claim that may be made by its manufacturer, is not guaranteed or endorsed by the publisher.
